# Understanding the relationship between slicing and measured fill density in material extrusion 3D printing towards precision porosity constructs for biomedical and pharmaceutical applications

**DOI:** 10.1186/s41205-020-00063-8

**Published:** 2020-04-25

**Authors:** Prashanth Ravi

**Affiliations:** Cincinnati, OH USA

**Keywords:** Fill density, Scaffold porosity/strength, 3D printing, Controlled drug release, Mathematical modeling, Tissue engineering

## Abstract

**Background:**

Fill density is a critical parameter affecting the functional performance of 3D printed porous constructs in the biomedical and pharmaceutical domain. Numerous studies have reported the impact of fill density on the mechanical properties, diffusion characteristics and content release rates of constructs. However, due to the way in which slicing toolpath calculations are performed, there is substantial deviation between the measured and slicing fill density for relatively small sized constructs printed at low fill densities (high porosities). The purpose of the current study was to investigate this discrepancy using a combination of mathematical modeling and experimental validation.

**Methods:**

The open source slicer Slic3r was used to 3D print 20 mm × 20 mm × 5 mm constructs at three identified slicing fill density values, 9.58%, 20.36% and 32.33% (exact values entered into software), in triplicates. A mathematical model was proposed to accurately predict fill density, and the measured fill density was compared to both the predicted as well as the slicing fill density. The model was further validated at two additional slicing fill densities of 15% and 40%. The total material within the construct was analyzed from the perspective of material extruded within the beads as well as the bead to bead interconnects using the predictive model.

**Results:**

The slicing fill density deviated substantially from measured fill density at low fill densities with absolute errors larger than 26% in certain instances. The proposed model was able to predict fill density to within 5% of the measured fill density in all cases. The average absolute error between predicted vs. measured fill density was 3.5%, whereas that between slicing vs. measured fill density was 13%. The material extruded in the beads varied from 86.5% to 95.9%, whereas that extruded in the interconnects varied from 13.5% to 4.1%.

**Conclusions:**

The proposed model and approach was able to predict fill density to a reasonable degree of accuracy. Findings from the study could prove useful in applications where controlling construct fill density in relatively small sized constructs is important for achieving targeted levels of functional criteria such as mechanical strength, weight loss and content release rate.

## Background

Three-dimensional (3D) printing, also known as additive manufacturing or rapid prototyping, enables the fabrication of complex geometries without the need for any part-specific tooling or dies [1, 2]. 3D printing refers to a family of layered manufacturing processes including the process known as Material Extrusion (ME). In ME, a thermoplastic filament is melt-extruded through a nozzle in pre-defined paths to build the object in a layer-by-layer fashion [3]. The software used to generate these pre-defined paths, commonly referred to as toolpaths, is called a slicer. A Standard Tessellation Language (STL) file to be fabricated is typically imported into a slicer, and the toolpaths for printing the model are generated by the slicer based on settings specified by the user. Slic3r [4], a ME slicer developed in 2011, is used extensively by ME printing users because it allows customization of many characteristics in the printed part [5–10].

3D printing has been employed in the pharmaceutical domain such as for fabricating tailored dosage forms for drug delivery purposes owing to its precise and repeatable nature [11]. 3D printing also allows the fabrication of highly porous and complex products and the ability to personalize dosages as well as combine several drugs into a single pill [12]. The drug is released into the solvent as the bulk material slowly erodes, and the release rate and dosage can be controlled using print parameters such as the fill density and the number of shells [13, 14]. For instance, Goole et al. reported that increasing the fill density could drastically prolong the release of the drug [15]. The FDA has expressed growing interest in this area with the first 3D printed drug approved in August 2015 with Aprecia Pharmaceutical’s immediate release tablet for epilepsy SPRITAM [16].

Several research groups have studied the correlation between slicing parameters such as bead width, air gap and fill density on the mechanical properties of the printed construct [17–20]. One of the less investigated correlations is between slicing fill density and actual or measured fill density of the printed structure. Relatively smaller constructs printed at low fill densities result in significant errors in the slicing fill calculations in extrusion 3D printing due to the nature of the toolpathing calculations explained later in the manuscript. In the biomedical domain the porosity (1 – fill density ratio) of 3D printed constructs is often a critical parameter that determines ease of nutrient diffusion into and waste disposal away from the scaffold [21]. Porosity has also been shown to be strongly correlated with the compressive strength [22–25]. Fu et al. found the compressive strength of scaffolds to increase from 40 MPa to 140 MPa with an increase in fill density from 20% to 40% [25]. Williams et al. also found the compressive strength of scaffolds to increase from 2 MPa to 3.2 MPa with an increase in fill density from 45% to ~ 62% [24]. Because porosity represents the void fraction, it can be precisely controlled only through the accurate prediction/control of the material deposited within the matrix. Many researchers have reported measured porosities and some have attempted to model porosity accurately with success, but to the author’s knowledge none have compared their measurements or predictions to slicing fill density or slicing porosity [26–31]. Recently several research groups have investigated the fabrication of tablets using extrusion 3D printing [13, 32–38]. Goyanes et al. found that the amount of Fluorescein released from 3D printed tablets was influenced by the fill density [32]. Yang et al. found the release of Ibuprofen to be significantly affected by the fill density of the 3D printed tablet [37]. Thus, understanding how slicing parameters affect actual fill density is important in applications where the printed construct fill density strongly influences characteristics such as mechanical strength, weight loss and drug release rate.

There were two major factors behind selecting Slic3r for generating toolpaths and comparing fill density calculations with the model proposed in this study. Firstly, Slic3r assumes an oblong cross-section of beads in its calculations which is quite close to both published experimental and simulation results [39–42]. Secondly, Slic3r was developed in 2011 and has been used extensively by 3D printing researchers in the biomedical community and outside who develop their own in-house 3D printers and bioprinters [5–7, 37, 43–45]. Therefore, findings from this study using Slic3r could potentially serve helpful to a larger user base.

**Fig. 1:**
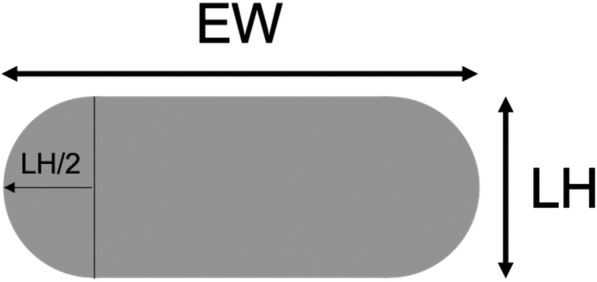
Schematic showing the cross-sectional shape of an extruded bead assumed by Slic3r. The EW and LH can be set in the software prior to slicing to adjust the bead aspect ratio as desired

Slic3r assumes an oblong cross-sectional shape for extruded beads as seen in Fig. 1 with the overall width equal to EW (Extrusion Width) and the height equal to LH (Layer Height). The semi-circular ends are each assumed to have a radius equal to half the LH.

Based on this model the bead cross-sectional area (Extr_area_) is calculated as follows:

1 $$ {Extr}_{area}=\left( EW- LH\right)\times LH+\pi /4\times {LH}^2 $$

**Fig. 2:**
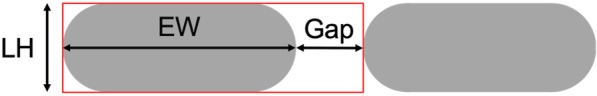
Schematic showing the cross-sectional view of two adjacent beads within a single layer. The area used for fill related calculations is boxed in red

Slic3r calculates the Gap distance (gap between adjacent beads within a layer) using a cross-sectional area-based rather than true layer volume-based model as seen in Fig. 2. Based on the area-based definition of fill density we get:

2 $$ \frac{Slic3r\  Fill\ Density\%}{100}=\frac{Extr_{area}}{\left( EW+ Gap\right)\times LH} $$

Based on a fill density percent value in the software by a user, Slic3r then calculates the Gap between adjacent extrusions within a layer using the following re-arranged form of Eq. 2:

3 $$ Gap=\frac{\frac{Extr_{area}}{LH}}{\frac{Slic3r\  Fill\ Density\%}{100}}- EW $$

**Fig. 3:**
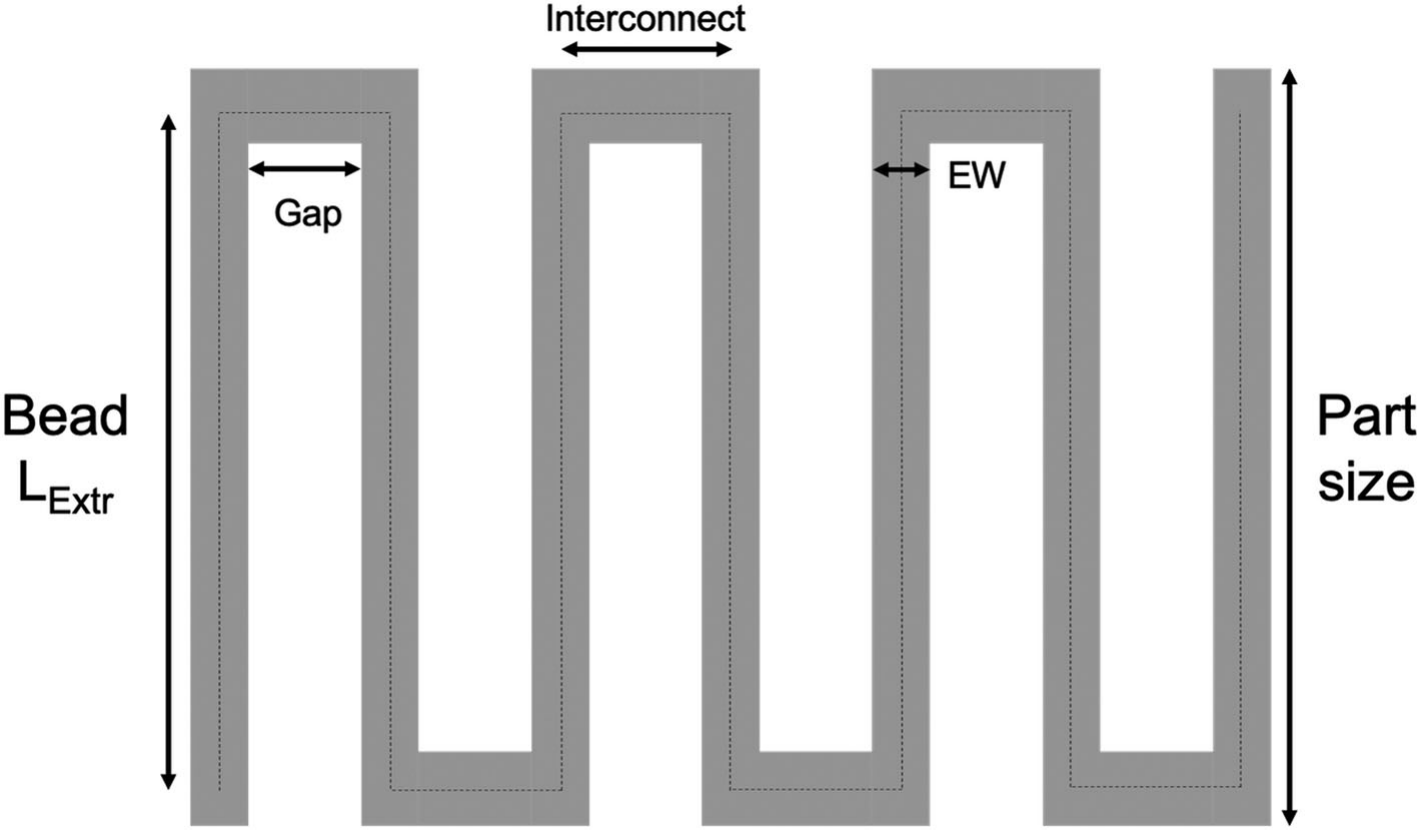
Top view schematic of a single printed layer showing the nozzle path (dotted line), Bead length (L_Extr_), Gap, Interconnect length, Extrusion Width (EW) and Part Size

The primary drawback of the area-based fill calculation aforementioned is that it relies on the assumption that the gap between beads is fully empty, which in reality is not the case because adjacent beads are joined by connecting beads spanning the gap as seen in Fig. 3 to connect them and preserve extruder pressure. However, the assumption is reasonable for constructs printed at high fill density, since the Gap size reduces with increasing fill density percent as seen in Eq. 3. However, this assumption results in significant errors for relatively small sized constructs printed at low fill density percent as seen in the manuscript.

The present study aims to study the correlation between slicing fill density and measured fill density of the printed part using the slicing software Slic3r. The study also presents a mathematical model to predict fill density to a reasonable degree of accuracy based on the actual toolpath generated by Slic3r. The methodology reported in this paper might prove useful in 3D printing biomedical scaffolds and pharmaceutical tablets with more targeted fill density which could in turn potentially result in more accurate mechanical properties and weight loss characteristics.

## Methods

### Proposed model for calculating fill density percent

The proposed model developed in this work includes the material extruded in the bead-to-bead interconnects in an effort to improve the agreement of predicted fill density compared to the measured density. The schematic in Fig. 3 represents the top view of a single printed layer showing L_Extr_ (length of extrusion from toolpath i.e. nozzle motion), EW and Gap distance. If the number of parallel beads (extrusions) in a single layer separated from each other by the center-to-center distance of Gap + EW is represented by n, then we can describe the predicted fill density based on the toolpath as:

4 $$ Predicted\ Fill\ Density\%=\frac{Extr_{area}\times {L}_{Extr}\times n+\left(\left[ Gap+ EW\right]\times {Extr}_{\mathrm{a} rea}\right)\times \left(n-1\right)}{Layer_{vol}}\times 100 $$

where *Layer*_*vol*_ = *L*_*Extr*_ × *L*_*Extr*_ × *LH* for each printed layer in the cuboidal construct. Substituting this in Eq. 4 yields:

5 $$ Predicted\ Fill\ Density\%=\left(\frac{Extr_{area}\times n}{L_{Extr}\times LH}+\frac{\left(\left[ Gap+ EW\right]\times {Extr}_{area}\right)\times \left(n-1\right)}{{L_{Extr}}^2\times LH}\right)\times 100 $$

Therefore, the predicted fill density percent is computed based on the parameters EW and LH set in the software, other parameters Extr_area_, Gap and Layer_vol_ calculated using the aforementioned equations while L_Extr_ and n are obtained from the Slic3r generated toolpath (G-code) file and Slic3r slice preview.

### Experimental setup

**Fig. 4:**
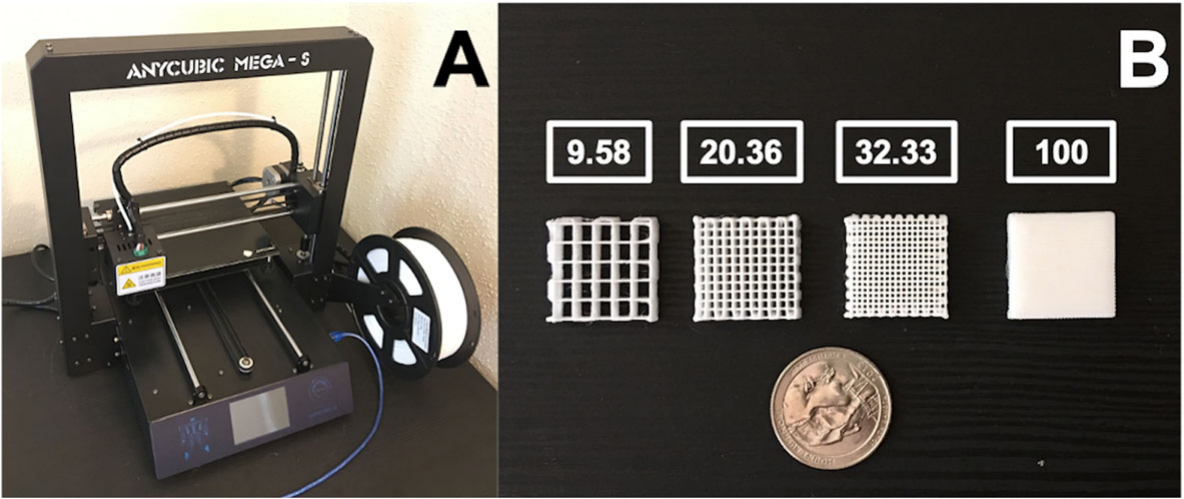
The 3D printer used for validating the predicted fill density percent calculations (**a**) and 20 × 20 × 5 mm^3^ samples (right) with the respective Slic3r fill density percent values in inset boxes shown with a quarter dollar coin (~ 24 mm diameter) for scale (**b**)

An Anycubic Mega-S 3D Printer (Anycubic, Commerce, CA, USA) was used for experimentally validating the predicted fill density percent calculations from Eq**.**4. Polylactic acid (PLA) filament branded as PLA+ 3D filament (Anycubic, Commerce, CA, USA) with diameter equal to 1.75 ± 0.02 mm and density of 1.26 g/cc was used. A temperature of 220 °*C*, print speed of 20 mm/sec, LH of 0.2 mm and EW of 0.4 mm were set in the slicer. The fill pattern was set to Rectilinear with 0/90 alternating fill angles and the number of perimeters/outlines was set to 0 (Fig. 4). The software package employed to generate toolpaths was Slic3r 1.2.9 [4] and Repetier-Host 1.0.2 (Hot-World GmbH & Co. KG, Willich, Germany) was used to communicate toolpaths to the printer. A 20 mm × 20 mm × 5 mm model was created in SolidWorks (Dassault Systemes Americas Corp., Waltham, MA, USA) and converted to the STL format. To ensure proper calibration of the extruder steps a 100% fill density was set in Slic3r and the imported 20 mm × 20 mm × 5 mm STL was printed in triplicates and weighed using a precision digital weighing scale (Newacalox 8028, Newacalox, USA).

**Fig. 5:**
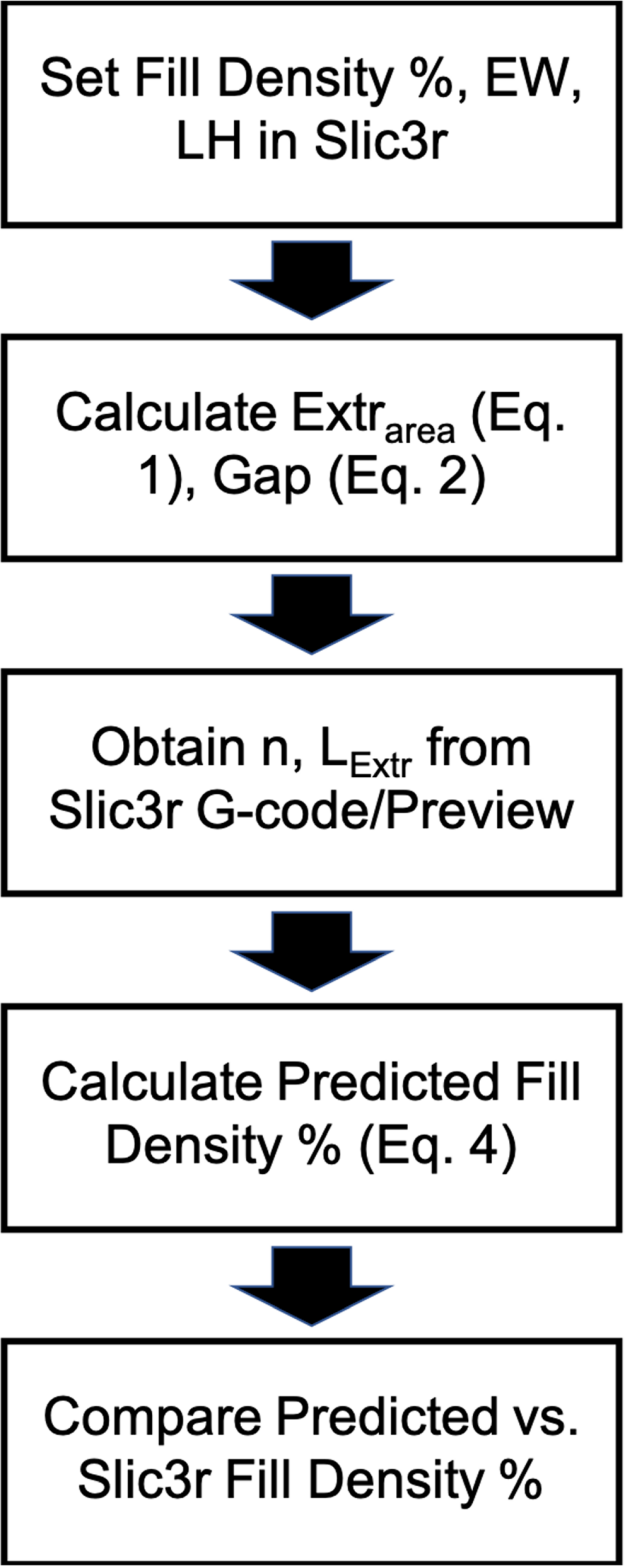
Flow chart showing the sequence of steps to be followed to calculate predicted fill density percent and compare against the Slic3r fill density percent

The number of extrusions per layer (n) in a 3D printed part is always an integer value, since partial extrusions are not generated in the toolpaths by slicing software. This implies that there is this additional source of error in the fill calculations arising from the discretization of the number of beads in addition to the ignored bead-to-bead interconnect extrusion. The aim of this study was to understand the effect of the latter variable, and therefore 3 fill density percent values were identified (close to the 10, 20 and 30% fill density values) through trial and error in the software such that n was very close to an integer value based on observations from the slicing preview i.e. the number of extrusions generated per layer. These fill density percent values were 9.58%, 20.36% and 32.33% resulting in 6, 12 and 18 extrusions, respectively. Reducing each of these fill density percent values even by 0.01 reduced the number of extruded beads per layer by 1, indicating that these fill density percent values resulted in the closest integer values for the number of extrusions. Such a trial and error approach was necessary because Slic3r performs an internal scaling of the part size before generating n. Generally, n is the rounded down integer value of the (Part size + Gap)/(EW + Gap) ratio [26]. However, it was observed that Slic3r performs an unknown adjustment of this ratio, and consequently the n observed in the sliced preview was slightly different than estimated using the ratio. The sequence of steps to obtain and compare predicted fill density percent with the Slic3r fill density percent can be found in Fig. 5. As seen, the procedure includes a combination of calculating quantities using equations in this manuscript as well as finding quantities from the G-code and Slic3r slice preview. Samples in triplicates were also fabricated at 15% and 40% Slic3r fill density to further validate applicability of the approach at additional values other than the three aforementioned.

All printed samples were weighed and measured along the XYZ axes using digital Vernier calipers. The predicted fill density percent was calculated using Eq. 4 based on the EW and LH set in the software and Gap was calculated using Eq. 3 (also verified from the Slic3r G-code). L_Extr_ was obtained from the G-Code co-ordinate data and n identified from the Slic3r preview. L_Extr_ was found to be 19.878 mm from the G-Code co-ordinate data. Measured fill density percent was calculated based on the sample weight and XYZ dimensions using Eq. 6, and predicted weight was calculated using Eq. 7. The absolute error percent values were always calculated relative to the measured quantity (measured weight or measured fill density percent) using Eq. 8.

6 $$ Measured\ Fill\ Density\%=\frac{\frac{Measured\ weight}{\rho }}{X\times Y\times Z}\times 100 $$

where *ρ* is the density of PLA (1.26 g/cc) and X, Y and Z are the respective dimensional measurements.

7 $$ Predicted\ Weight=\left({Extr}_{area}\times {L}_{Extr}\times n+\left(\left[ Gap+ EW\right]\times {Extr}_{area}\right)\times \left(n-1\right)\right)\times number\ of\ layers\times \rho $$

where the $$ number\ of\ layers=\frac{Part\ Z\  height}{LH}=\frac{5 mm}{0.2 mm}=25 $$

8 $$ Abs. Error\%=\frac{Abs\left( Predicted\ or\ Slic3r- Measured\right)}{Measured}\times 100 $$

## Results

The extruder calibration was verified based on the error between predicted vs. measured weight at 100% Slic3r fill density. At 100% fill density Slic3r overlaps adjacent beads by 0.043 mm as seen in the G-code and accurately calculated using Eq. 3**.** The error between predicted and measured weight at 100% Slic3r fill density was 0.2% on average which demonstrates excellent extruder calibration (Table 1). The measured weight was on average 1.1% larger than the fully dense weight on the other hand. The XYZ dimensions of the 15 samples were all quite close to the CAD design without any significant deviation (Table 2). The weights within each Slic3r fill density percent group were also highly consistent.

**Table 1:** Measured weight, predicted weight and absolute percent error for the 100% fill density samples

Sample	Slic3r Fill Density %	Fully Dense Weight (g)	Measured Weight (g)	Predicted Weight (g)	Abs. % Error (Predicted vs. Measured)	Abs. % Error (Dense vs. Measured)
1	100	2.520	2.560	2.548	0.5	1.1
2	100	2.520	2.551	2.548	0.1	1.1
3	100	2.520	2.550	2.548	0.1	1.1

**Table 2:** Measured weight and dimensional measurements for the samples printed at 5 fill density percent values in triplicates

Sample	Slic3r Fill Density %	X (mm)	Y (mm)	Z (mm)	Measured Weight (g)
1	9.58	20.08	19.99	4.98	0.322
2	9.58	20.08	20.00	4.90	0.323
3	9.58	20.08	19.91	4.86	0.320
1	20.36	20.08	20.13	4.94	0.603
2	20.36	20.10	20.13	4.92	0.599
3	20.36	20.05	20.14	4.97	0.602
1	32.33	20.02	20.03	4.90	0.881
2	32.33	20.04	20.04	4.93	0.875
3	32.33	19.98	20.00	4.90	0.872
1	15.00	19.98	19.94	4.92	0.404
2	15.00	20.05	19.97	4.93	0.407
3	15.00	20.04	19.90	4.92	0.411
1	40.00	19.93	19.99	4.92	1.057
2	40.00	19.91	19.96	4.93	1.063
3	40.00	19.94	20.01	4.92	1.056

The error between the Slic3r vs. measured fill density percent was substantial at 9.58% (Table 3). The average error was 26.1% whereas the error between predicted vs. measured fill density percent was 3.8%. The error (Slic3r vs. measured) was also substantial at the intermediate 20.36% Slic3r fill density percent setting, with an average of around 14.8% compared to the predicted vs. measured average error of 2.4%. The predicted fill density percent was also in good agreement with the measured fill density percent at the 15% and 40% Slic3r fill density percent values as seen in Table 3. Overall, the average Slic3r vs. measured fill density percent error was 13%, whereas the average predicted vs. measured fill density percent error was 3.5% which is nearly 4-fold less. The errors for the predicted vs. measured fill density percent at all five Slic3r fill density percent values were less than 5%, which is at a much more acceptable level. Overall, the measured fill density percent was about 1.5–3.5% larger than the Slic3r fill density percent. Table 4 shows how the relative contribution of material extruded in the bead-to-bead interconnects reduces with increasing fill density from 13.51% at 9.58% Slic3r fill density to 4.11% at 40% Slic3r fill density.

**Table 3:** Measured and predicted fill density along with errors for the samples printed at 5 fill density percent values in triplicates

Sample	Slic3r Fill Density %	Measured Fill Density %	Predicted Fill Density %	Abs. % Error (Predicted vs. Measured)	Abs. % Error (Sli3r vs. Measured)
1	9.58	12.78	12.46	2.5	25.1
2	9.58	12.99	12.46	4.3	26.5
3	9.58	13.15	12.46	4.7	26.7
1	20.36	23.97	23.30	2.8	15.1
2	20.36	23.88	23.30	2.4	14.7
3	20.36	23.81	23.30	2.1	14.5
1	32.33	35.58	34.03	4.4	9.2
2	32.33	35.07	34.03	3.0	7.8
3	32.33	35.34	34.03	3.7	8.5
1	15.00	16.36	15.88	2.9	8.3
2	15.00	16.36	15.88	2.9	8.3
3	15.00	16.62	15.88	4.5	9.8
1	40.00	42.80	41.21	3.7	6.5
2	40.00	43.06	41.21	4.3	7.1
3	40.00	42.69	41.21	3.5	6.3

**Table 4:** Breakdown of the predicted material extruded per layer within parallel beads and bead-to-bead interconnects along with their relative contributions

Slic3r Fill Density %	No. of Beads	Total Bead Length (mm)	Total Bead-to-bead Interconnect Length (mm)	Bead Contribution %	Interconnect Contribution %
9.58	6	119.27	18.64	86.49	13.51
20.36	12	238.54	19.29	92.52	7.48
32.33	18	357.80	18.78	95.01	4.99
15.00	8	159.02	16.66	90.52	9.48
40.00	22	437.32	18.75	95.89	4.11

## Discussion

The aim of the current study was to understand the difference between fill density percent set in Slic3r vs. the fill density percent measured in the actual part. The printing process was highly repeatable as seen from the consistency in weights between samples printed at the same fill density percent values in Tables 1 and 2. During extruder calibration, the overlap between adjacent beads generated within Slic3r is necessary to fill all the void spaces between the oblong shaped cross-section of beads and create a true 100% dense construct. With an overlap of 0.043 mm the center-center distance between beads became 0.4–0.043 = 0.357 mm. Slic3r extrudes plastic during this 0.357 mm travel from current bead to the next bead as well and that is why the measured weight is larger than 2.520 g, which is the estimated weight for a fully dense 20 mm × 20 mm × 5 mm model printed at 100% fill density. Because the predicted weight calculation includes the bead-to-bead interconnecting extrusions in the calculation, it can be seen that it is much closer to the measured weight (0.2% average error) than the fully dense weight (1.1% average error). The low error between predicted and measured weight helped verify extruder calibration as well as establish confidence in the predictive model for weight.

For samples printed at 9.58%, 20.36%, 32.33% as well as at 15% and 40% the weights were also consistent across samples at each Slic3r fill density percent as seen in Table 2, which demonstrates good repeatability in printing. The XYZ dimensions were quite close to the original design with the most deviation observed along the Z. This likely occurred because of the relatively large gaps (0.493 mm to 3.327 mm) between adjacent beads relative to EW that could have caused the extrusions in subsequent layers to slightly sag. As expected, the changes in weights did not correlate one to one with the changes in the Slic3r fill density percent. For instance, the average measured weight of the constructs printed at 100% Slic3r fill density was 2.554 g, whereas the average measured weight at 9.58% was 0.322 g. Similarly, it can be seen that the average of the sample weights at 20.36% Slic3r fill density was 1.87 times the average of the sample weights at 9.58%, whereas the ratio of the respective Slic3r fill density percent values was 2.13.

At low fill densities it was observed that the error between slicing fill density percent and measured fill density percent was substantially high (> 25%), but the predicted fill density percent was able explain the measured fill densities to well within 5% error. This difference was primarily due to the extruded material between extrusions (the center to center distance between adjacent beads or interconnects) being ignored in the slicer calculations (Table 4). This error becomes significant at low infill density since the gap distance between adjacent beads increases, and therefore the amount of material extruded in the bead-to-bead interconnects increases as a relative portion of the total material extruded. The average error in Table 3 reduces from more than 26.1% at 9.58% Slic3r fill density to an average error of 6.6% at 40.00% Slic3r fill density, which shows the rapidly decreasing proportion of the material extruded in the bead-to-bead interconnects compared to the total material extruded (Table 4).

In addition to the three identified Slic3r Fill Densities of 9.58%, 20.36% and 32.33%, the predicted fill density percent was in close agreement with measured fill density percent at 15% and 40% Slic3r fill density as well thus demonstrating applicability of the methodology laid out to other arbitrarily low fill density values. To the author’s knowledge, this is the first study to investigate the relationship between measured and slicing fill density using the open source slicer Slic3r. Several studies have reported data regarding measured porosity percent (100 – measured fill density percent) and some have tried to predict porosity and compare it against the measured porosity. Too et al. modeled porosity in terms of the EW, LH, n, Gap and part size and observed good agreement between measured and predicted porosity for porosities under 70% which correspond to fill densities over 30% [26]. However, they assumed a rectangular cross-section of the beads and also did not compare their results with the fill density percent set in the software since they were using a closed source software. Woodfield et al. modeled porosity in terms of n, bead diameter, number of layers and part size and found reasonable agreement between measured and predicted porosity in the 55–88% range, but their measured porosity was in most cases 7–8% lower than that predicted [27]. This was likely the case because they did not consider the material extruded in the bead-to-bead interconnects in their model, and they also assumed the bead to have a perfect circular cross-section. However, they too did not compare the measured and predicted porosities against the slicing fill density/porosity. Zein et al. modeled porosity in terms of n, bead diameter and the number of layers but found the predicted values to deviate significantly (> 10%) in many instances in either direction when compared to the measured porosity [28]. The reason for this was likely because they assumed a cylindrical cross-section for the beads and ignored the bead-to-bead interconnects, but they also did not compare the measurements against the sliced fill density. Armillotta et al. modeled porosity using a unit cell approach in terms of the bead thickness and Gap assuming a square cross section for beads, but found significant deviation compared to measured values which they attributed to measurement inaccuracies [29]. Shor et al. modeled the porosity in terms of the gap, bead diameter and sine of the bead angle, but did not report any measured porosities for their samples [31]. Hattiangadi et al. modeled the porosity in terms of EW, LH and Gap assuming an elliptical cross-section for the beads, but found substantial differences compared to experimental measurements which they attributed to micro porosity in their structures [30].

The approach laid out in the current study allows researchers to predict the actual fill density to a reasonable degree of accuracy, particularly in low fill density (high porosity) scenarios, based on slicing parameters EW, LH and fill density percent set in the open source slicer Slic3r. Being able to accurately predict the fill density before actually printing the part is quite beneficial, and could also allow researchers to adjust slicing parameters for achieving a target fill density in the part. For instance, one potential method to achieve a target fill density in the construct would be to estimate density using the predicted fill density percent model and then reduce the extrusion multiplier in the slicer to achieve the desired density. As mentioned earlier, this is vital in biomedical applications since the porosity (100 – measured fill density percent) is such a crucial parameter affecting the diffusion of nutrients into and the removal of waste out of printed constructs in vitro and in vivo, which consequently affects the growth and proliferation of cells seeded within the construct. Researchers have also found strong correlation of porosity with the compressive strength of constructs, so being able to accurately predict porosity (from accurate prediction of fill density) could result in more accurate mechanical properties.

This study has some limitations. First, the predictive model for fill density percent in its current form is only applicable to cuboidal parts, since otherwise the bead-to-bead interconnects might end up being curves or other geometries based on the contour shape for that layer. Next, the study assumes a fixed oblong cross-sectional shape for extruded beads, but the actual deposition process is complex and there are certainly deviations from this shape based on the slicing parameters, underlying substrate, direction of nozzle travel etc. Furthermore, the difference in error between predicted fill density percent vs. measured fill density percent and Slic3r fill density percent vs. measured fill density percent becomes more and more insignificant at higher fill densities, since the material extruded in the bead-to-bead interconnects becomes smaller relative to the total material extruded. Moreover, the methodology laid out is best applicable to relatively small sized constructs since the contribution from material extruded in the bead-to-bead interconnects in inversely proportional to the square of bead lengths as seen in the second term of the right hand side in Eq. 5. Finally, the model and findings are presently only applicable to the 0/90-degree fill pattern.

## Conclusions

At low fill densities the measured fill density was found to deviate substantially from the slicing fill density with average absolute errors greater than 26% in certain instances for a 20 mm × 20 mm × 5 mm cuboidal construct. The predictive fill density percent model in the study estimated fill density to well within 5% of the measured fill density percent values at all five low fill density settings, since the model is based on the actual slicer (Slic3r) toolpath and takes into account the material extruded in the bead-to-bead interconnects. The approach presented in this study could be used to predict construct fill density with reasonable accuracy based on slicing parameters in Slic3r. The methodology could prove useful in biomedical and pharmaceutical applications requiring accurate control of fill density for relatively small sized constructs to meet targeted functional criteria such as compressive strength and content release rate.

## Data Availability

All data generated and/or analyzed during the current study are available from the corresponding author on reasonable request.

## Abbreviations

EW: Extrusion width
LH: Layer height
Extr_Area_: Cross-sectional area of extrusions
L_Extr_: Length of extrusion from the G-code
n: Number of beads (extrusions) in a single layer
Gap: Distance between the closest ends of adjacent extrusions
Layer_Vol_: Volume of a single layer
X: Measured part size along the X-axis
Y: Measured part size along the Y-axis
Z: Measured part size along the Z-axis
*ρ*: Density of PLA
